# Factors Associated With Cancer Treatment Delay Among Patients Diagnosed With COVID-19

**DOI:** 10.1001/jamanetworkopen.2022.24296

**Published:** 2022-07-28

**Authors:** Samyukta Mullangi, Emeline M. Aviki, Yuan Chen, Mark Robson, Dawn L. Hershman

**Affiliations:** 1 Department of Medicine, Memorial Sloan Kettering Cancer Center, New York, New York; 2 Department of Surgery, Memorial Sloan Kettering Cancer Center, New York, New York; 3 Department of Biostatistics, Memorial Sloan Kettering Cancer Center, New York, New York; 4 Department of Medicine, Columbia University Medical Center, New York, New York

## Abstract

**Importance:**

The COVID-19 pandemic led to disruptions in delivery of cancer treatments; factors associated with treatment delay among patients with cancer who contract COVID-19 need further characterization.

**Objective:**

To assess the associations of patient factors, social determinants of health, severity of COVID-19, and timing of COVID-19 diagnosis with the risk of treatment delay.

**Design, Setting, and Participants:**

This prospective cohort study was conducted from March 2020 through July 2021 at 60 academic and community medical practices in the United States. Participants included patients with any cancer diagnosis who were scheduled for treatment and contracted COVID-19. Data were analyzed in February 2022.

**Exposure:**

Positive test result for SARS-CoV-2.

**Main Outcomes and Measures:**

The main outcomes were treatment delay, defined as more than 14 days between the date originally planned for treatment and the date of initiation of therapy, or discontinuation of therapy. Multivariable analyses were used to assess outcomes.

**Results:**

A total of 3028 patients (1470 patients [49%] aged ≥65 years; 1741 [58%] women) were included in the registry. With 962 of 2103 patients (46%) experiencing anticancer drug delay or discontinuation, delays were higher among Black patients compared with White patients (odds ratio [OR], 1.87; 95% CI, 1.40-2.51), Hispanic or Latino patients compared with non-Hispanic or Latino patients (OR, 1.91; 95% CI, 1.34-2.72), patients with 2 or more comorbidities compared with patients with 0 to 1 (OR, 1.23; 95% CI, 1.00-1.53), patients with metastatic disease rather than locoregional disease (OR, 1.63; 95% CI, 1.29-2.05), and patients who experienced COVID-19 complications compared with those who did not (OR, 1.52; 95% CI, 1.24-1.86). Residing in an area with a higher proportion of residents reporting Hispanic or Latino ethnicity (OR, 0.76; 95% CI, 0.60-0.95) and contracting COVID-19 later in the pandemic, compared with those who were infected in March to June 2020, (eg, January to March 2021: OR, 0.38; 95% CI, 0.26-0.53) were associated with lower likelihood of drug therapy delay. A total of 95 of 202 patients (47%) experienced delay or discontinuation of radiation treatment, with having 2 or more comorbidities associated with delay (OR, 2.69; 95% CI, 1.20-6.20). Higher local-area median household income was associated with lower likelihood of radiation treatment delay (OR, 0.41; 95% CI, 0.17-0.94). There were 89 of 125 patients (71%) who experienced surgical treatment delay, and delays were higher among patients in the South compared with those in the Midwest (OR, 9.66; 95% CI, 2.14-52.3). Interestingly, patients with 2 or more comorbidities, compared with those with 0 to 1, experienced lower likelihoods of surgical treatment delay (OR, 0.26; 95% CI, 0.07-0.88).

**Conclusions and Relevance:**

Our findings suggest that individual patient factors, social determinants of health, and COVID-19 severity and diagnosis date were associated with exacerbated health disparities during the pandemic in regards to cancer treatment delay.

## Key Points

**Question:**

What are the factors associated with cancer treatment delay among patients with test results positive for SARS-CoV-2?

**Findings:**

In this cohort study using data from 3028 patients in a large COVID-19 oncology data registry, multiple different patient demographic factors, such as race and ethnicity, underlying primary malignant neoplasm (diagnosis and extent of spread), multimorbidity, geographic location, receipt of COVID-19 vaccine, severity of COVID-19 infection, and timing of COVID-19 diagnosis, were associated with delays in cancer treatment.

**Meaning:**

These findings suggest that some health disparities may have been exacerbated during the pandemic as an effect of cancer treatment delay.

## Teaser

This cohort study assesses the associations of patient factors and social determinants of health with the risk of treatment delay in patients with cancer who contract COVID-19.

## Introduction

As a result of system- and patient-level factors related to the COVID-19 pandemic, cancer care has frequently been delayed or altered. For example, recent data showed that half of European breast centers altered systemic treatments during the pandemic and one-fifth of patients experienced delay in radiation therapy.^1^ Professional societies quickly released guidance to amend care guidelines during a time of great uncertainty, prior to vaccines and other therapies.^2,3,4,5,6^ These guidelines helped clinicians prioritize patients with higher-risk disease to avoid treatment delays and suggested alternatives, such as beginning systemic therapy first to permit surgical treatment to be delayed.^7,8^ Changes to conventional treatment plans have subsequently been studied for associations with outcomes.^9,10^ Such studies help researchers identify cancers and clinical settings in which delays are not associated with overall survival, as well as situations in which delays in treatment truly are harmful.^11^ However, these studies have largely assessed the impact of the pandemic among patients with cancer who did not contract COVID-19 themselves.

There has been a paucity of data on how oncologists altered care for patients who had a positive test result for SARS-CoV-2. Furthermore, much of the data accumulated over the course of the pandemic was contributed by academic medical centers or single institutions, and therefore may not be generalizable. In this study, we use the American Society of Clinical Oncology (ASCO) COVID-19 Oncology Data Registry, a prospective cohort study launched at the beginning of the pandemic with 60 participating community and academic medical practices, to describe the characteristics, outcomes, and factors associated with treatment delay among patients with cancer who contracted COVID-19. We hypothesized that patient demographic factors, primary malignant neoplasm, social determinants of health (SDH), severity of COVID-19, and timing of COVID-19 diagnosis would be associated with delays in care.

## Methods

This cohort study was not deemed to be human participants research and was therefore considered exempt from review and informed consent by the Western and Memorial Sloan Kettering Cancer Center institutional review boards. This report follows the Strengthening the Reporting of Observational Studies in Epidemiology (STROBE) reporting guideline for observational studies.

### Data Source

The ASCO COVID-19 Oncology Data Registry is a prospective database that was launched in March 2020. The specifics regarding the practices and data collection have been previously described.^12^ Participating practices registered patients who had a positive SARS-CoV-2 test result and met any of the following criteria: initiating treatment for any new diagnosis of cancer, clinically evident cancer receiving either anticancer treatment or supportive care only, or disease-free but receiving adjuvant therapy within 1 year after surgical resection. The registry collects information on demographics, risk factors, cancer diagnosis and treatment status, SARS-CoV-2 infection (including symptoms, treatments, hospitalizations, and long-term sequelae), and mortality. Information about race and ethnicity at the participant level are self-reported by patients at time of inclusion into their clinics’ electronic health record. Race was classified as American Indian or Alaska Native, Asian, Black, or White and ethnicity was classified as Hispanic or Latino, not Hispanic or Latino, other (including individuals who did not report Hispanic or Latino or non–Hispanic or Latino ethnicity), or unknown.

From date of initial data entry, follow-ups on the patient’s clinical status were solicited at 1, 2, 3, 6, 9, 12, 18, and 24 months. Birth date, home zip code, and practice name were used to link data from the same patient across follow-ups. Practices also submitted data retrospectively for patients who experienced illness earlier in the pandemic.

Five SDH variables, including census tract–level racial and ethnic make-up (ie, population reporting White race or Hispanic ethnicity), median household income, insurance level, and education level, were analyzed directly by ASCO and provided to researchers as quartiles to preserve deidentification of patient data. These variables were estimated by matching patient zip code to the 2018 American Community Survey data, the largest household survey conducted by the US Census Bureau, and assigning quartiles based on all US zip codes.^13^ Electronic health record data were abstracted and manually entered into a secure REDCap survey form (Vanderbilt University).^14,15^

### Covariates

Age was dichotomized to age groups younger than 65 years or 65 years and older. Patient comorbidities were categorized as 0 to 1 and 2 or more, based on clinically relevant chronic conditions (the complete list includes alcohol use disorder, chronic supplemental oxygen needed, cirrhosis, congestive heart failure, coronary artery disease, dementia, diabetes, hepatitis, history of solid organ transplant, HIV/AIDS, hypertension, immunosuppressed due to noncancer-related treatment [eg, use of corticosteroids >10 mg/d prednisone, chemotherapy, immunosuppressive agents for solid organ transplant or for autoimmune disease], inflammatory bowel disease, pulmonary disease, kidney disease, and systemic autoimmune disease). COVID-19–related complications were defined as having developed a pneumonia, been hospitalized, needed supplemental oxygen, or needed antiviral therapies specific to SARS-CoV-2. COVID-19 vaccine status was obtained from patients’ baseline assessments. *International Statistical Classification of Diseases and Related Health Problems, Tenth Revision (ICD-10)* diagnoses were grouped into categories owing to overall small numbers. The 5 SDH variables, which were made available to researchers as quartiles, were dichotomized as above and below the median, with standard median splits.

### Outcomes

We evaluated all patients who were documented as having cancer therapy (anticancer drug therapy, surgical treatment, or radiation therapy) scheduled at the time of entry into the registry. Time of entry into the registry was time of first positive SARS-CoV-2 test result. We created separate cohorts to individually assess delays in anticancer drug therapy, surgical treatment, and radiation therapy.

Treatment delay was defined as more than 14 days between date originally planned for treatment and rescheduled date of initiation of therapy. Discontinuation of therapy was classified as a delay.

### Statistical Analysis

Univariate and multivariable analyses were conducted to investigate the association between patient characteristics and treatment delay. For univariate analyses, we summarized the median and IQRs of the continuous variables and frequency distributions for categorical variables. Wilcoxon rank sum test was used to evaluate whether continuous variables differed by therapy delay status. Pearson χ^2^ test (Fisher exact test for sparse categorical variables) was used to investigate whether the categorical variables differed by therapy delay status. We conducted logistic regression to examine the multivariable associations of treatment delay with patient characteristics. We removed patient covariates with sparse categories to help with model convergence. All hypothesis tests were 2-sided with a Type I error of 5%.

We performed a robustness check by running a multivariable regression using only significant covariates in univariate analysis. We treated the quartiles of SDH covariates as separate categories and dichotomized them around the median (the third and fourth quartiles vs the first and second quartiles).

Data cutoff occurred at July 30, 2021. All analyses were conducted using R statistical software version 4.1.1. Data were analyzed in February 2022.

## Results

### Patient Characteristics

At the time of data analysis, 3028 patients (1470 patients [49%] aged ≥65 years; 1741 [58%] women) were included in the ASCO COVID-19 data registry. Of these, 2103 patients had scheduled anticancer drug therapy, 125 patients had scheduled surgical treatment, and 202 had scheduled radiation therapy. Overall, 2275 (75%) patients had a solid tumor malignant neoplasm and 1062 patients (47%) had metastatic disease. The most common cancer diagnoses were breast and hematologic cancers. Baseline clinical factors by delay status are shown in Table 1.

**Table 1.** Characteristics of Patients Included in the Analysis From the ASCO Registry

Categories	No. (%) (N = 3028)
Age ≥65, y	1470 (49)
Gender	
Men	1283 (42)
Women	1741 (58)
Race	
American Indian or Alaska Native	192 (6)
Asian	247 (8)
Black	420 (14)
White	2169 (72)
Ethnicity	
Hispanic or Latino	297 (10)
Not Hispanic or Latino	2451 (81)
Other or unknown^a^	280 (9)
Smoking history	
No	1527 (53)
Yes	1375 (47)
Solid tumor	2275 (75)
Stage (solid tumors only)	
Early	1213 (53)
Metastatic	1062 (47)
Cancer groups	
Breast	736 (24)
Gastrointestinal and neuroendocrine	487 (16)
Genitourinary	394 (13)
Hematological malignancy	712 (24)
Lung	352 (12)
Other	347 (11)
BMI	
<18.5	66 (2)
18.5-24.9	801 (27)
25-29.9	945 (32)
>30	1142 (39)
Comorbidity index	
0-1	2051 (68)
≥2	977 (32)
COVID-19 vaccine at baseline assessment	
Yes	136 (14)
No	840 (86)
COVID-19 complications	1295 (43)
Zip code–level SDH, median (IQR)	
Population reporting Hispanic ethnicity, %^b^	3 (2-4)
Population reporting White race, %^c^	2 (1-2)
Median household income^d^	3 (2-4)
Population with only a high school diploma, %^e^	2 (1-3)
Population age <65 y with no health insurance, %^f^	3 (2-3)
US Region	
Midwest	897 (30)
Northeast	464 (15)
South	1403 (46)
West	261 (9)
COVID-19 diagnosis time period	
March-June 2020	600 (20)
July-September 2020	606 (20)
October-December 2020	1226 (40)
January-March 2021	438 (14)
April-July 2021	158 (5)

Abbreviations: BMI, body mass index (calculated as weight in kilograms divided by height in meters squared); SDH, social determinants of health.

^a^ Includes individuals who identified as an ethnicity other than Hispanic or Latino or non–Hispanic or Latino.

^b^ Quartiles for zip code–level percentage of population reporting Hispanic ethnicity: 1, <0.7%; 2, 0.7% to 3.1%; 3, 3.2% to 9.5%; 4, ≥9.5%.

^c^ Quartiles for zip code–level percentage of population reporting White race: 1, <77.3%; 2, 77.4% to 92.1%; 3, 92.2% to 97.4%; 4, ≥97.5%.

^d^ Quartiles for zip code–level median household income: 1,<$43 125; 2, $43 125 to $54 047; 3, $54 048 to $68 446; 4, >$68 446.

^e^ Quartiles for zip code–level percentage of population with only a high school diploma: 1, <25.5%; 2, 25.5% to 33.7%; 3, 33.8% to 41.2%; 4, ≥42.2%.

^f^ Quartiles for zip code–level percentage of population with no health insurance: 1, <4.8%; 2, 4.8% to 8.8%; 3, 8.9% to 14.7%; 4, ≥14.8%.

In the total cohort, there were 192 American Indian or Alaska Native patients (6%), 247 Asian patients (8%), 420 Black patients (15%), and 2169 White patients (72%). Additionally, 297 patients (10%) identified as Hispanic or Latino ethnicity and 2451 patients (81%) identified as not Hispanic or Latino. Overall, 1375 patients (47%) had a documented smoking history, either current or previous cigarette users. There were 2051 patients (68%) with 0 to 1 comorbidities, and 977 patients (32%) with 2 or more comorbidities. A total of 945 patients (32%) had body mass index (BMI; calculated as weight in kilograms divided by height in meters squared) of 25 to 29.9; 1142 patients (39%) had BMI greater than 30, and 66 patients (2%) had BMI less than 18.2 (Table 1).

By the data cutoff point of July 30, 2021, a total of 136 patients (14%) had received a COVID-19 vaccine, which reflects the temporality of the survey instrument. Time period of enrollment is available in Table 1. A total of 1295 patients (43%) were identified as having experienced any COVID-19–related complication.

### Missing Data

Of 3028 patients included in the ASCO COVID-19 data registry, 2 had unknown age, 4 had unknown gender, 260 had unknown ethnicity, 126 had unknown smoking status, 3 had missing residence region, 1062 had unknown metastatic status, and 83 had at least 1 unknown SDOH variable. Unknown ethnicity, smoking status, and metastatic status were coded as a separate category so that more patients could be included in the analysis. Thus, less than 3% of patients were deleted from the analysis owing to missing data.

### Treatment Delays

#### Chemotherapy Delay

We found that 962 of 2103 patients (46%) of patients had a delay of at least 14 days or discontinuation of anticancer drug treatment (Figure). A multivariable logistic regression evaluating factors associated with anticancer drug delay found that delays were higher among Black patients compared with White patients (odds ratio [OR], 1.87; 95% CI, 1.40-2.51), and among Hispanic or Latino patients compared with non-Hispanic or Latino patients (OR, 1.91; 95% CI, 1.34-2.72) (Table 2). Residing in an area with a higher proportion of residents reporting Hispanic ethnicity was associated with lower likelihood of delay (OR, 0.76; 95% CI, 0.60-0.95). Compared with patients with 0 to 1 comorbidities, having 2 or more comorbidities was associated with delay in treatment (OR, 1.23; 95% CI, 1.00-1.53). Having metastatic disease, compared with local or regional disease, (OR, 1.63; 95% CI, 1.29-2.05), and having any COVID-19 complications, compared with having none, (OR, 1.52; 95% CI, 1.24-1.86) were associated with delay. Compared with having breast cancer, having gastrointestinal and neuroendocrine cancer, hematologic malignant neoplasms, lung cancer, or other cancers were associated with anticancer drug delay (Table 2). Conversely, compared with patients who contracted COVID-19 during the initial outbreak of the pandemic (March-June 2020), receiving a COVID-19 diagnosis later in the pandemic was associated with lower likelihood of delay (eg, January to March 2021: OR, 0.38; 95% CI, 0.26-0.53). Receipt of COVID-19 vaccine was associated with a lower likelihood of delay (OR, 0.48; 95% CI, 0.29-0.78).

**Figure.**
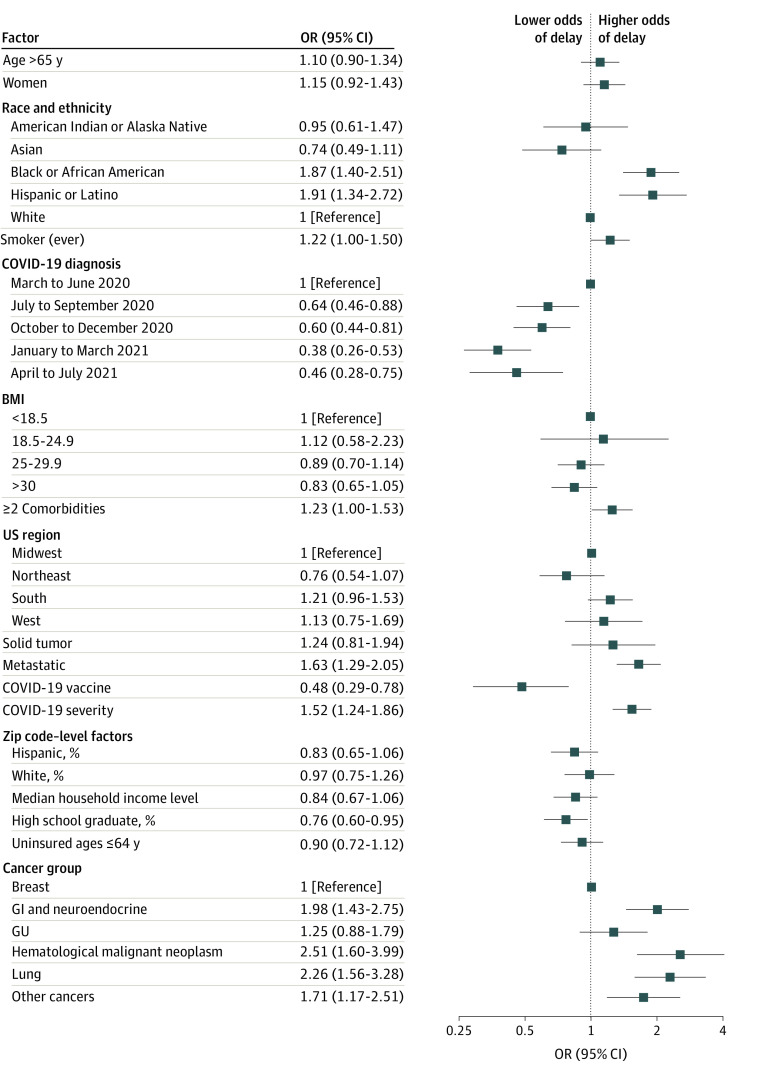
Subgroup Analysis of Anticancer Drug Therapy Delay Among Patients With COVID-19 Zip code–level variables are binary variables, defined as higher than median vs lower than median. BMI indicates body mass index, calculated as weight in kilograms divided by height in meters squared; GI, gastrointestinal; GU, genitourinary; OR, odds ratio.

**Table 2.** Multivariable Logistic Regression Model for Factors Associated With Treatment Delay

Characteristic	OR (95% CI)		
	Drug therapy (n = 2103)	Radiation (n = 202)	Surgical treatment (n = 125)
Age ≥65 y	1.10 (0.90-1.34)	1.43 (0.68-2.99)	0.87 (0.25-2.97)
Gender			
Men	1 [Reference]	1 [Reference]	1 [Reference]
Women	1.15 (0.92-1.43)	1.20 (0.52-2.76)	3.68 (0.85-18.4)
Race			
American Indian or Alaska Native	0.95 (0.61-1.47)	1.10 (0.27-4.47)	0.14 (0.01-2.65)
Asian	0.74 (0.49-1.11)	1.89 (0.51-7.25)	0.49 (0.09-2.66)
Black	1.87 (1.40-2.51)^a^	1.06 (0.33-3.47)	1.00 (0.17-6.72)
White	1 [Reference]	1 [Reference]	1 [Reference]
Ethnicity			
Hispanic or Latino	1.91 (1.34-2.72)^a^	NA	1.63 (0.24-15.4)
Not Hispanic or Latino	1 [Reference]	NA	1 [Reference]
Other^b^	1.04 (0.28-3.63)	NA	0.19 (0.00-9.01)
Unknown	1.31 (0.90-1.91)	NA	1.09 (0.12-13.8)
Smoking history			
No	1 [Reference]	1 [Reference]	NA
Yes	1.22 (1.00-1.50)	1.84 (0.83-4.19)	NA
Solid Tumor	1.24 (0.81-1.94)	NA	NA
Stage			
Nonmetastatic	1 [Reference]	NA	NA
Metastatic	1.63 (1.29-2.05)^a^	NA	NA
Cancer groups			
Breast	1 [Reference]	1 [Reference]	1 [Reference]
GI and neuroendocrine	1.98 (1.43-2.75)^a^	0.42 (0.12-1.44)	8.41 (1.46-58.9)^a^
GU	1.25 (0.88-1.79)	0.24 (0.07-0.76)^a^	1.07 (0.21-5.66)
Hematological malignant neoplasm	2.51 (1.60-3.99)^a^		
Lung	2.26 (1.56-3.28)^a^	0.26 (0.07-0.85)^a^	2.76 (0.20-82.4)
Other	1.71 (1.17-2.51)^a^	0.44 (0.14-1.38)	2.32 (0.43-14.5)
BMI			
<18.5	1.12 (0.58-2.23)	0.29 (0.04-2.03)	NA
18.5-24.9	1 [Reference]	1 [Reference]	NA
25-29.9	0.89 (0.70-1.14)	1.10 (0.43-2.84)	NA
>30	0.83 (0.65-1.05)	0.74 (0.30-1.82)	NA
Comorbidity index			
0-1	1 [Reference]	1 [Reference]	1 [Reference]
≥2	1.23 (1.00-1.53)^a^	2.69 (1.20-6.20)^a^	0.26 (0.07-0.88)^a^
COVID-19 vaccine at baseline assessment			
No	1 [Reference]	1 [Reference]	1 [Reference]
Yes	0.48 (0.29-0.78)^a^	0.24 (0.03-1.48)	0.20 (0.01-3.05)
COVID-19 complications	1.52 (1.24-1.86)^a^	1.83 (0.82-4.15)	1.09 (0.26-4.79)
Zip code–level SDH			
Population reporting Hispanic ethnicity, %	0.83 (0.65-1.06)	1.15 (0.47-2.81)	0.93 (0.66-4.72)
Population reporting White race, %	0.83 (0.65-1.06)	0.94 (0.36-2.48)	1.25 (0.30-5.88)
Median household income	0.84 (0.67-1.06)	0.41 (0.17-0.94)^a^	0.68 (0.17-2.71)
Population with only a high school diploma, %	0.76 (0.60-0.95)^a^	0.92 (0.39-2.15)	0.48 (0.13-1.81)
Population (age <65 y) with no health insurance, %	0.90 (0.72-1.12)	0.56 (0.25-1.25)	0.71 (0.20-2.36)
US region			
Midwest	1 [Reference]	1 [Reference]	1 [Reference]
Northeast	0.76 (0.54-1.07)	1.00 (0.31-3.13)	1.16 (0.14-9.93)
South	1.21 (0.96-1.53)	1.40 (0.61-3.22)	9.66 (2.14-52.3)^a^
West	1.13 (0.81-1.94)	1.04 (0.21-4.96)	1.74 (0.22-14.9)
COVID-19 diagnosis time period			
March-June 2020	1 [Reference]	1 [Reference]	1 [Reference]
July-September 2020	0.64 (0.46-0.88)^a^	1.12 (0.33-3.84)	0.09 (0.01-0.61)^a^
October-December 2020	0.60 (0.44-0.81)^a^	1.34 (0.46-4.01)	0.42 (0.04-3.37)
January-March 2021	0.38 (0.26-0.53)^a^	0.40 (0.11-1.33)	0.07 (0.00-0.91)^a^
April-July 2021	0.46 (0.28-0.75)^a^	0.55 (0.08-3.23)	0.20 (0.01-8.48)

Abbreviations: BMI, body mass index (calculated as weight in kilograms divided by height in meters squared); GI, gastrointestinal; GU, genitourinary; NA, not available; OR, odds ratio; SDH, social determinants of health.

^a^ *P* < .05.

^b^ Includes individuals who identified as an ethnicity other than Hispanic or Latino or non–Hispanic or Latino.

#### Radiation Therapy Delay

We found that 95 of 202 patients (47%) had a delay of at least 14 days or discontinuation of radiation therapy. Summary results from multivariable logistic regression showed that the factors associated with radiation therapy delay included having 2 or more comorbidities compared with 0 to 1 comorbidities (OR, 2.69; 95% CI, 1.20-6.20). Compared with breast cancer, patients with genitourinary and lung cancer had lower risk of radiation therapy delay (Table 2). Residing in an area with higher median household income was associated with lower likelihood of delay (OR, 0.41; 95% CI, 0.17-0.94).

#### Surgical Treatment Delay

We found that 89 of 125 patients (71%) had a delay of at least 14 days or discontinuation of surgical treatment. Summary results from multivariable logistic regression showed that the factors associated with surgical treatment delay included living in the South, compared with living in the Midwest, (OR, 9.66; 95% CI, 2.14-52.3) and having gastrointestinal and neuroendocrine cancer, compared with breast cancer, (OR, 8.41; 95% CI, 1.46-58.9) (Table 2). Having 2 or more comorbidities, compared with 0 to 1 comorbidities, (OR, 0.26; 95% CI, 0.07-0.88) and being diagnosed with COVID-19 in July to September 2020 (OR, 0.09; 95% CI, 0.01-0.61) or January to March 2021 (OR, 0.07, 95% CI, 0.00-0.91), rather than March to June 2020, were associated with lower likelihood of delay.

## Discussion

In this large prospective cohort study, we found that multiple patient factors, such as race and ethnicity, underlying primary malignant neoplasms (ie, diagnosis and extent of spread), multimorbidity, geographic location, receipt of COVID-19 vaccine, severity of COVID-19, and timing of COVID-19 diagnosis, were associated with delays in cancer treatment. Interestingly, we only found associations between a few social determinants of health variables and likelihood of treatment delay. These findings provide insight into the state of cancer care during the most significant public health emergency of this era.

When the COVID-19 pandemic first emerged in 2020, it imposed a great deal of hardship and confusion on physicians and health care systems, which often did not have contingency plans ready for a global infectious disease outbreak. As a result, nonstandardized adaptation strategies quickly emerged to triage patients with high risk and either reduce or reallocate services. One global systematic review that identified 38 different categories of delays and disruptions reported that clinician- or system-related variables, such as medicine stockouts or shortages of devices, personal protective equipment, and laboratorial or imaging tests, were the most frequently reported structural or process-related factors associated with delay.^16^ Several single-institution reports and surveys of physicians revealed that there were more treatment delays among patients infected with SARS-CoV-2 compared with patients who were not infected.^17,18^ Our analysis is the first study, to our knowledge, to identify systemic delays or cancellations in cancer treatment for a cohort of patients who were all diagnosed with SARS-CoV-2 infection.

The data from our study suggest that disparity borne of treatment delays in the setting of a positive SARS-CoV-2 test result may also be one of the reasons contributing to overall poor outcomes among already vulnerable populations. Vulnerable populations include people who are economically disadvantaged, racial and ethnic minority groups (including Asian, Black, and Hispanic individuals), people without health insurance, children in low-income families, elderly adults, people experiencing homelessness, individuals with HIV, and individuals with other chronic health conditions, including severe mental illness.^2,19^ This classification may also include rural residents, who often encounter barriers to accessing health care services. The vulnerability of these individuals is enhanced by race, ethnicity, age, gender, and factors such as income, insurance coverage (or lack thereof), and absence of a usual source of care.^1,4,5,6,7,8^ Their health and health care problems intersect with social factors, including housing, poverty, and inadequate education. Our findings are in line with previous data that have shown persistent disparities in COVID-19–related outcomes in subgroups of disadvantaged and minority patients and populations. In its most recent data release (October 5, 2021), the Centers for Disease Control and Prevention shared that Black people make up a similar share of cases relative to their share of population (12%) but account for a higher share of deaths comparatively (14% vs 12%).^20^ Furthermore, age-standardized data show that American Indian and Alaska Native, Black, and Hispanic people are 2-fold more likely to die from COVID-19 than their White counterparts.^20^ The reasons for these disparities are myriad, including gaps in vaccination rates, spread of the virus initially to urban areas (which include more racially and ethnically diverse populations), and risk of infection concentrated among essential workers, who are disproportionately members of racial and ethnic minority communities.^21^

While we did not observe an increased propensity for treatment delay among patients with hematologic malignant neoplasms in our study compared with those with solid tumors overall, we did observe a greater association of hematologic malignant neoplasm with treatment delay compared with the breast cancer group. Other studies have identified an increased risk for COVID-19 mortality among patients with hematologic malignant neoplasms, linking this to virus-specific properties, but not assessing the association with treatment delays.^22,23,24,25^ To the extent that treatment delays may be contributing to overall increased mortality in this subgroup, special attention must be paid to how treatment delay or discontinuation protocols developed by institutions may be inadvertently exacerbating adverse outcomes in a high-risk group.

In our study, we noted that compared with individuals who contracted COVID-19 during the initial outbreak of the pandemic from March to June 2020, contracting COVID-19 later in the pandemic was associated with a lower likelihood of treatment delay and reduction in delays. A 2022 analysis on the ASCO COVID-19 registry by Mileham et al^12^ found that before June 2020, the main reason for testing for SARS-CoV-2 was COVID-19 symptoms (74% of tests). After June 2020, fewer patients were tested for symptoms (49% of tests; *P* < .001) and more patients were tested for routine oncology care (35% of tests after vs 7% of tests before; *P* < .001). Mortality rates also corresponded accordingly with these protocols. In the study by Mileham et al,^12^ patients diagnosed with COVID-19 before June 2020 had a 30-day mortality rate of 20% (95% CI, 14%-25%) compared with 13% (95% CI, 8%-18%) for those diagnosed after June 2020. This indicates that changes in COVID-19 management, and correspondingly, patient outcomes, emerged as clinicians developed an increasing understanding of the disease and made more nuanced accommodations to improve access to care.

We also wish to highlight the finding that receipt of vaccination against COVID-19 was associated with lower likelihood of anticancer drug delay. Data show that the COVID-19 vaccination is particularly important in patients with cancer for a variety of reasons—patients with weakened immune systems are at high risk for severe complications from COVID-19, and vaccines decrease the risk of hospitalization and death from COVID-19 even among individuals with cancer.^26,27,28^ Yet, the data also show that most patients undergoing cancer treatment produce antibodies at a slower rate than individuals without cancer.^29^ Given this understanding, data such as ours are helpful to adjudicate the vaccine’s efficacy in improving outcomes in this high-risk population.

Finally, the ability of the COVID-19 pandemic to uncover and exacerbate underlying disparities borne from SDH has been well-documented.^30^ Yet, we did not find consistent associations between the 5 SDH variables and treatment delays in our data. We attribute this discrepancy to a few different factors, chief among them being that the SDH data were drawn from the American Community Survey and linked by zip code to patient data, rather than data abstracted directly from the electronic health record, which means that our conclusions are, at best, associative. The SDH data are from 2018, and are not representative of the real-time status of patients’ social, professional, and financial health. Finally, the small sample sizes, particularly in the radiation and surgical therapy cohorts, also limited our ability to draw more robust and reliable conclusions from the data.

### Limitations

This study has some limitations. First, the ASCO registry contains limited information about practice factors, which may have been associated with treatment delay during the pandemic. The patients seen within the ASCO participating practices may not represent the general population; furthermore, we do not have information on whether the treatment delays had any associations with outcomes. Additionally, the study only enrolled patients who had test results positive for SARS-CoV-2; therefore, we did not have any information on control patients with cancer but without COVID-19 who were treated in the same practices and who may or may not have experienced delay. We were unable to explicitly distinguish the cause of delay, so it is not clear how much of the delay was directly related to symptoms of COVID-19, isolation requirements, or structural factors. As this observational registry relied on manual inputting of data from ambulatory oncology clinics, there was a large amount of missing data, which ultimately may have affected several of our analyses. Although there was sufficient sample size for some comparisons, certain subgroup analyses, particularly in the radiation and surgical therapy categories, were underpowered. Additionally, the low sample sizes necessitated dichotomization of key variables, such as SARS-CoV-2 infection severity and treatment delay. As the registry continues to recruit patients and increase in size, we anticipate that future analyses may be able to be more nuanced in assessment.

## Conclusions

In this cohort study, multiple patient factors, such as race and ethnicity, underlying primary malignant neoplasms, multimorbidity, geographic location, receipt of COVID-19 vaccine, severity of COVID-19, and timing of COVID-19 diagnosis, were associated with delays in cancer treatment. Quantification of the impact of COVID-19 on practice management can provide insight to health system leaders and public health authorities about the extent of disruptions in care, and differential impacts on vulnerable and/or disadvantaged populations. We hope that the knowledge generated through studies like ours can help inform proposals for well-targeted strategies to improve planning, preparation, patient support, and resource allocation during the ongoing COVID-19 pandemic and future national health emergencies.
